# Cardiac Tamponade in Down's Syndrome Associated With Hypothyroidism: An Uncommon Presentation

**DOI:** 10.7759/cureus.59023

**Published:** 2024-04-25

**Authors:** Rabia Iqbal, Joshua A Wilson, Hnin Nadi Linn, Ahmad Taimoor Bajwa, Kanchan Devi, Pradeep Kumar Devarakonda

**Affiliations:** 1 Medicine, The Brooklyn Hospital Center, Brooklyn, USA; 2 Internal Medicine, The Brooklyn Hospital Center, Brooklyn, USA; 3 Internal Medicine, Cardiovascular Disease, Icahn School of Medicine at Mount Sinai, New York, USA

**Keywords:** pericardial effusion and cardiac tamponade, critical care, transthoracic echocardiogram, hypothyroidism, down's syndrome

## Abstract

Down syndrome often coincides with hypothyroidism, a condition that may lead to pericardial effusion (PE), though cardiac tamponade remains an infrequent complication. Cardiac tamponade is an emergency that requires immediate diagnosis and treatment. Here, we present a case of a patient who presented to the emergency department (ED) with Down syndrome associated with hypothyroidism and underwent immediate pericardiocentesis and pericardial window placement. A 52-year-old male, with a history of Down's syndrome and hypothyroidism, presented to the ED complaining of shortness of breath and chest pain. He had previously been diagnosed with PE. On examination, he exhibited average heart rate, low blood pressure, decreased heart sounds, and jugular venous distention, with no murmur or frictional rub. Initial investigations revealed normal sinus rhythm on EKG but an enlarged cardiac silhouette on chest X-ray. Laboratory tests showed elevated C-reactive protein and sedimentation rate, suggestive of inflammation, while arterial blood gas showed compensated respiratory alkalosis. Thyroid-stimulating hormone (TSH) was elevated. Despite supplemental oxygen, the patient's condition worsened, prompting a bedside ultrasound revealing cardiac tamponade. A cardiology consultation recommended immediate transfer for treatment. At a different hospital, pericardiocentesis was performed, followed by the placement of a pericardial window to prevent recurrence. Follow-up imaging showed improvement in pleural effusion and resolution of cardiac tamponade. The patient's symptoms improved, and he was discharged with regular follow-up. Down's syndrome is a chromosomal disorder characterized by the trisomy of chromosome 21. It is associated with various cardiac complications. Such patients have an elevated risk of PE due to a variety of reasons, such as viral infections, hypothyroidism, or autoimmune diseases. Although PE has been found, the incidence of cardiac tamponade has rarely been reported. The pathogenesis of PE in hypothyroidism is due to the leakage of fluids from the capillaries and the build-up of fluid in the pericardial space. The treatment of PE is treating hypothyroidism with thyroxine. In rare cases like ours, when the patient develops cardiac tamponade, the patient often needs pericardiocentesis. Our patient had to undergo pericardial window placement, as well to prevent recurrent symptoms. In conclusion, this case report sheds light on the occurrence of cardiac tamponade in a patient with Down's syndrome and hypothyroidism, a relatively rare complication that necessitates prompt recognition and intervention. Through this report, we emphasize the importance of considering cardiac tamponade in the differential diagnosis of patients with Down's syndrome presenting with symptoms suggestive of cardiovascular compromise.

## Introduction

Down syndrome is one of the most chromosomal anomalies, characterized by an additional copy of chromosome 21. It is often associated with various endocrine abnormalities, the most common of which is hypothyroidism. The estimated prevalence of hypothyroidism in Down's syndrome is nearly 0.12% [1]. Hypothyroidism can sometimes cause pericardial effusion (PE), which refers to fluid accumulation in the pericardial sac surrounding the heart. PE is familiar with the presence of myxedema in hypothyroidism, but its incidence in the absence of myxedema is rare. Studies have shown that the incidence of PE in hypothyroidism is 3-6% [2]. If not diagnosed and treated timely, it can lead to cardiac tamponade, which is a life-threatening condition where fluid accumulation compresses the heart and impairs its function. The literature reporting the incidence of PE and cardiac tamponade in Down's syndrome associated with hypothyroidism is limited. Only a few reports have reported this finding.

We have presented such a case that was observed and progressed to cardiac tamponade, despite being on treatment with levothyroxine.

## Case presentation

A 52-year-old male, with a past medical history of Down's syndrome and hypothyroidism on levothyroxine, presented to the ED with complaints of shortness of breath and chest pain. In the past, he had been evaluated for the same reasons and was found to have asymptomatic PE. 

On physical examination, the patient had normal heart rate and blood pressure, decreased heart sounds on auscultation, and jugular venous distention. He had no murmur or frictional rub. The rest of the physical exam was unremarkable.

**Figure 1:**
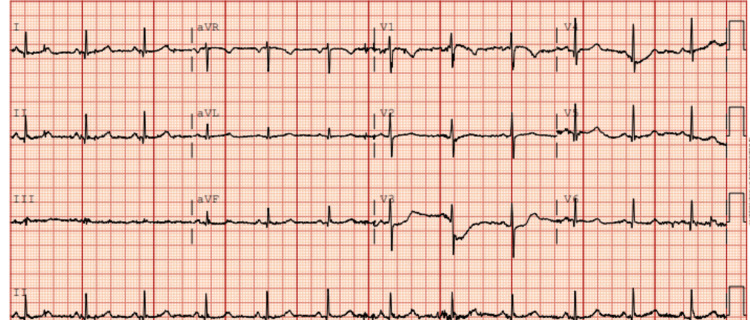
Initial EKG of the patient showing normal sinus rhythm

Initial EKG (Figure 1) showed normal sinus rhythm with a normal heart rate of 72. The blood pressure was 125/86 mmHg on admission.

**Figure 2:**
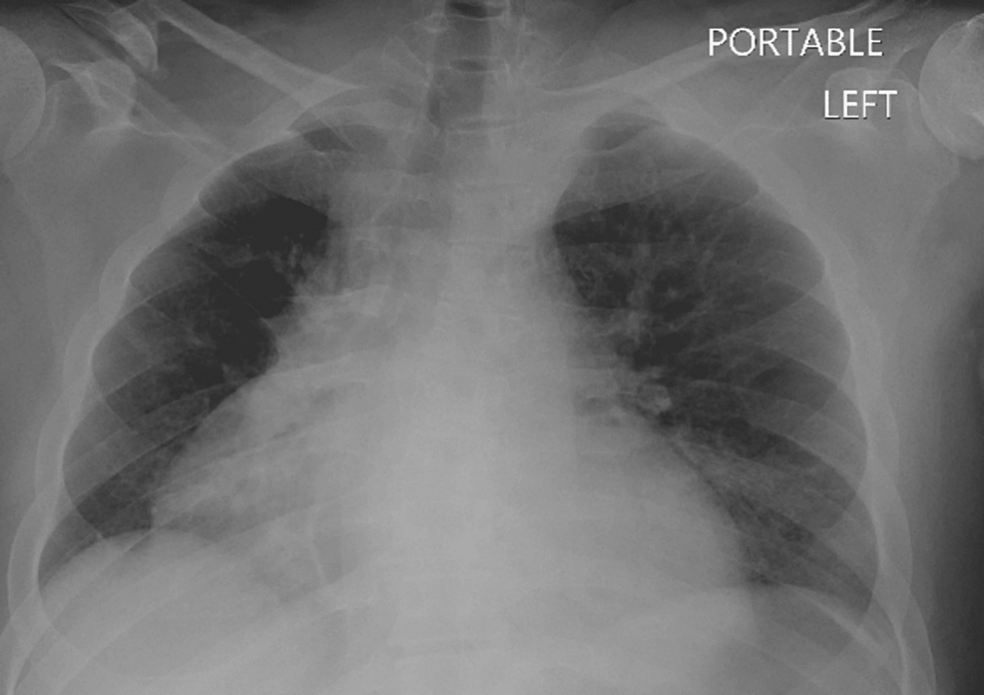
Initial chest X-ray showing the enlarged heart

The patient was placed on supplemental oxygen due to shortness of breath with no improvement. CXR showed an enlarged cardiac silhouette (Figure 2). 

**Figure 3:**
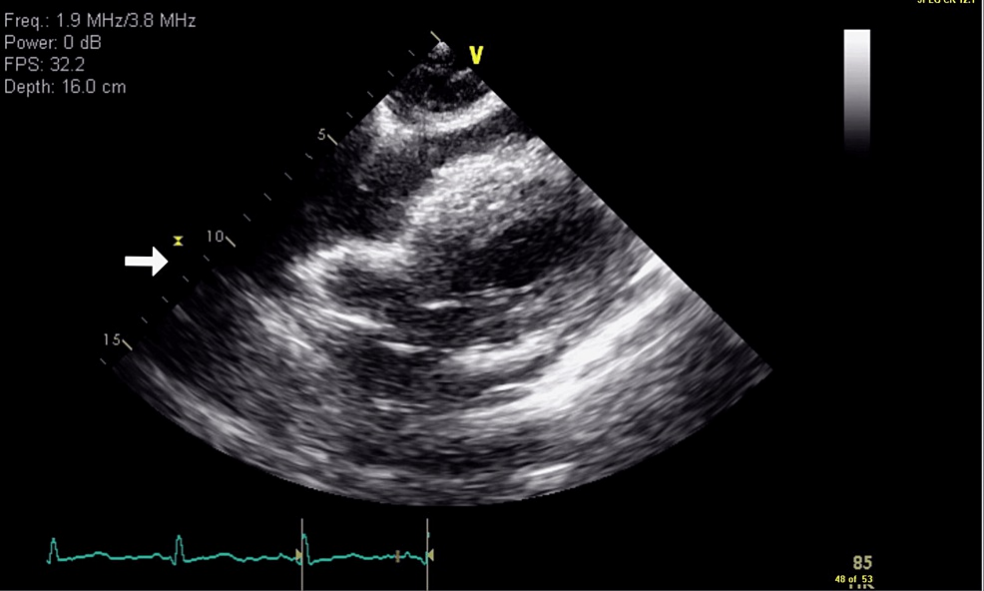
Transthoracic echocardiogram showing cardiac tamponade

Initial labs (Figure 3) showed the following results.

**Table 1:** Initial lab results of the patient

Labs Tests	Results	Reference Range
Hemoglobin	14.5	13.8 to 17.2 grams per deciliter (g/dL)
White cell count	5.0	4.5 to 11.0 × 109/L
Platelets	413	130-400 K/cmm
Sodium	135	136-145 mmol/L
Potassium	4.9	3.5-5.1 mmol/L
Creatinine	0.9	0.7-1.3 mg/dL
Blood urea nitrogen	12	7-25 mg/dL
C-reactive protein	35.48	<5 mg/L
Sedimentation rate	64	0-15 mm/hr

**Figure 4:**
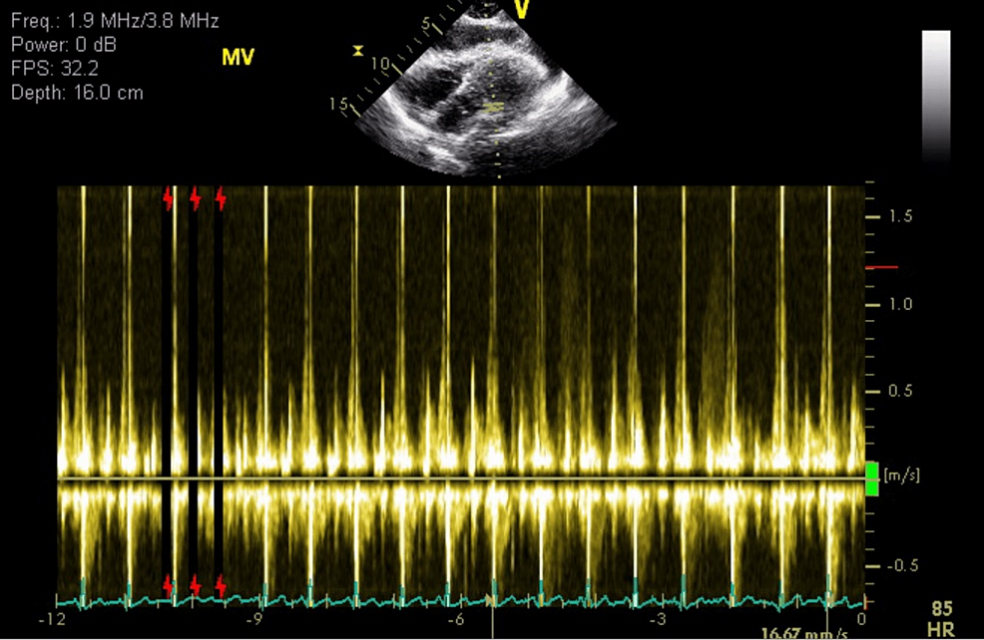
Respiratory inflow velocity of the mitral valve showing more than 20% drop with inspiration

Arterial blood gas showed a pH of 7.47, pCO2 of 40, pO2 of 80, bicarbonate of 29, and oxygen saturation of 97%. The thyroid stimulating hormone (TSH) was 10. Further history revealed that the patient was not compliant with his thyroid medications. When the patient's symptoms continued to worsen, a bedside ultrasound was performed that revealed cardiac tamponade. It was also confirmed with official echocardiogram (Figures 3-4).

Cardiology was consulted, and they recommended transferring the patient for immediate treatment. The patient was transferred to a different hospital, where he underwent pericardiocentesis, and a pericardial window was placed to prevent recurrent symptoms. 

**Figure 5:**
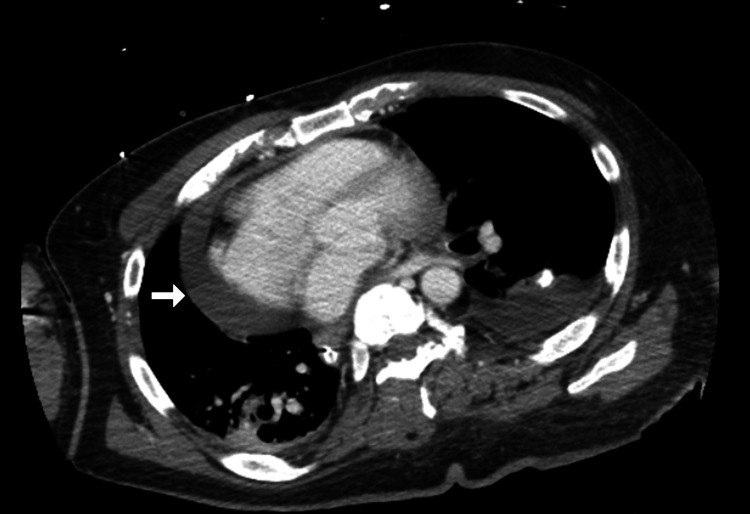
CT scan of the chest after the procedure

CT chest (Figure 5) repeated showed improvement in pleural effusion from before and no signs of cardiac tamponade.

The symptoms of shortness of breath improved, and he was discharged with regular follow-ups.

## Discussion

Down's syndrome is caused by trisomy 21 and is the most common chromosomal disorder. PE in Down's syndrome with hypothyroidism is uncommon. Most of the time, the effusion resolves by medications [3]. A study performed to evaluate the prevalence of PE in Down's syndrome showed that PE in Down's syndrome was 28% compared to those without Down's syndrome [4]. However, slow fluid accumulation can rarely result in cardiac tamponade [5], like in our case.

The pathogenesis of PE in Down's syndrome is not entirely understood. It can occur for various reasons, such as viral infection, hypothyroidism, and autoimmune diseases such as celiac disease [6]. Hypothyroidism causes increased permeability of albumin in the pericardial capillaries, which causes a decrease in the colloid osmotic pressure gradient between the pericardium and pericardial space, resulting in fluid accumulating in the pericardial space [7]. Our patient had chronic PE, but it converted into cardiac tamponade, requiring urgent treatment.

A literature review has shown a few case reports of the incidence of pericardial disease in patients with Down's syndrome (Table 2).

**Table 2:** Review of case reports found in the literature ASD: autism spectrum disorder

Cases	Authors	Patients Characteristics	Cardiac Tamponade	Pericardial Effusion	Associated Features	Clinical Manifestation	Treatment
1	Perttunen et al. [8]	49 Y/ F	No	Yes	Hypothyroidism	Breathing problems and chest pain	Levothyroxine
2	Anah et al. [9]	16 Y/ F	No	Yes	hypothyroidism	Generalized body swelling	Levothyroxine
3	Buyukkale et al. [10]	2-week-old infant	Yes	Yes	Leukemia	Respiratory distress	Pericardiocentesis and pericardial tube replacement
4	Verma et al. [11]	20 Y/F	Yes	Yes	Tuberculosis	recurrent bouts of cyanosis, abdominal swelling, and pedal edema	Diuretics
5	Said et al. [5]	39 Y/M	No	Yes	Hypothyroidism	Fatigue, palpitations, and bouts of cyanotic lips	Thyroxine
6	Sharaf El-Dean et al. [12]	26 Y/M	Yes	Yes	Hypothyroidism	Exertional shortness of breath, orthopnea, paroxysmal nocturnal dyspnea	Pericardial window
7	Falasco et al. [13]	14 month old	Yes	Yes	Transient abnormal myelopoiesis	Hypothermia (34.5°C), jaundice, and persistence of leukocytosis with blasts	Pericardiocentesis
8	Aleem et al. [14], Case 1	3 year old	No	Yes	ASD	Asymptomatic	Conservative management
9	Aleem et al. [14], Case 2	2.5-year-old female	Yes	Yes	ASD	Upper respiratory symptoms, decreased activity level, and shortness of breath over	Pericardiocentesis

As the symptoms of hypothyroidism and Down's syndrome are similar, such as fatigue, weight gain, and memory problems, the diagnosis of PE is often challenging. Due to a delay in diagnosis, it can sometimes lead to cardiac tamponade. Patients who develop PE need to undergo serial evaluations with echocardiography to evaluate the severity of the disease.

Hypothyroidism should be considered as one of the differentials when unexplained PE is found. The formation of effusion is slow, as the pericardial sac can stretch in response to the accumulation of the growing fluid. Due to this, patients often do not have symptoms, and diagnosis of PE can be missed [15]. When the fluid accumulates rapidly, the pericardial sac cannot collect the increased fluid, leading to cardiac tamponade. Cardiac tamponade is a medical emergency, and diagnosis depends on clinical symptoms and echocardiogram findings.

The clinical symptoms of cardiac tamponade include the triad of hypotension, jugular venous distention, and tachycardia. However, patients who have hypothyroidism present with bradycardia and typical vital signs, which can make the diagnosis even more difficult [16], as in our case. When the diagnosis of tamponade is suspected, echocardiography is the test used to confirm the diagnosis. It helps us visualize the presence and size of pericardial fluid. When the patients have tamponade, during diastole, the right atrium and right ventricle may appear to collapse due to the external pressure exerted by the PE seen on the echocardiogram, which confirms the diagnosis.

Some studies have shown that massive PE in children with Down's syndrome associated with hypothyroidism resolved with medical treatment without the need for pericardiocentesis [17]. The primary treatment for PE is levothyroxine. In the case of cardiac tamponade, the treatment includes the removal of fluids from the pericardial sac to reduce the pressure in the heart (18). To prevent recurrent episodes, some patients, like our case, need surgery to create a pericardial window to avoid the re-accumulation of fluid [18]. Once the patient is hemodynamically stable, treatment of underlying causes, like hypothyroidism in our case, is also necessary to prevent further recurrence. Compliance with medications is essential and should be stressed.

There are limitations to our case. While our patient also had thyroid disorders, which have been linked as a cause of PE, we cannot definitively state the underlying cause of the patient's PE. Therefore, further evaluation of the underlying cause of PE is limited in our case. Due to individual variation in disease severity and comorbidities, findings and management strategies presented in this case may not apply to all patients with Down's syndrome and hypothyroidism. However, despite the limitations, our case does share similarities with other instances of PE in patients with Down's syndrome.

## Conclusions

Cardiac tamponade is often a medical emergency. Although rare, hypothyroidism in patients with Down's syndrome can be a cause. Delays in treatment can lead to poor outcomes for patients. Elucidating possible differential diagnoses in patients with Down's syndrome could help prevent delays in treatment or missed diagnoses. Through this case, we aim to highlight early recognition of PE through ultrasonography, which allows for prompt initiation of appropriate management. We hope that healthcare practitioners will also be aware of this so that prompt evaluation of patients with Down's syndrome and dyspnea can be accomplished. It is the hope of the authors that more cases like these can help draw attention to possible differential diagnoses in patients with Down's syndrome that might otherwise be missed or delayed in treatment.
